# NADPH Oxidase Mediates Oxidative Stress and Ventricular Remodeling through SIRT3/FOXO3a Pathway in Diabetic Mice

**DOI:** 10.3390/antiox11091745

**Published:** 2022-09-02

**Authors:** Jiuchun Qiu, Daiqi Liu, Pengsha Li, Lingling Zhou, Lu Zhou, Xing Liu, Yue Zhang, Meng Yuan, Gary Tse, Guangping Li, Tong Liu

**Affiliations:** 1 Tianjin Key Laboratory of Ionic-Molecular Function of Cardiovascular Disease, Department of Cardiology, Tianjin Institute of Cardiology, The Second Hospital of Tianjin Medical University, Tianjin 300211, China; 2 Department of Cardiology, Wenzhou People’s Hospital, No. 299 Guan Road, Wenzhou 325000, China

**Keywords:** NADPH oxidase, oxidative stress, diabetic cardiomyopathy

## Abstract

Oxidative stress and mitochondrial dysfunction are important mechanisms of ventricular remodeling, predisposed to the development of diabetic cardiomyopathy (DCM) in type 2 diabetes mellitus. In this study, we have successfully established a model of type 2 diabetes using a high-fat diet (HFD) in combination with streptozotocin (STZ). The mice were divided into three groups of six at random: control, diabetes, and diabetes with apocynin and the H9c2 cell line was used as an in vitro model for investigation. We examined the molecular mechanisms of nicotinamide adenine dinucleotide phosphate (NADPH) oxidase activation on mitochondrial dysfunction and ventricular remodeling in the diabetic mouse model. Hyperglycemia-induced oxidative stress led to a reduced expression of sirtuin 3 (SIRT3), thereby promoting forkhead box class O 3a (FOXO3a) acetylation in ventricular tissue and H9c2 cells. Reactive oxygen species (ROS) overproduction promoted ventricular structural modeling and conduction defects. These alterations were mitigated by inhibiting NADPH oxidase with the pharmaceutical drug apocynin (APO). Apocynin improved SIRT3 and Mn-SOD expression in H9c2 cells transfected with SIRT3 siRNA. In our diabetic mouse model, apocynin improved myocardial mitochondrial function and ROS overproduction through the recovery of the SIRT3/FOXO3a pathway, thereby reducing ventricular remodeling and the incidence of DCM.

## 1. Introduction

Diabetes mellitus is a chronic metabolic condition that affects hundreds of millions of individuals. According to the International Diabetes Federation (IDF), diabetes affects roughly 463 million people aged 20 to 79 worldwide in 2019, with a projected increase of 578 million by 2030 [1]. Diabetic cardiomyopathy (DCM) is defined as a specific disease in diabetic patients with cardiac structural abnormalities and dysfunction independent of uncontrolled hypertension, coronary artery disease, and congenital heart disease [2]. The Framingham Heart Study reported that diabetic people have twice the risk of heart failure (HF) in men and five times in women as nondiabetic patients [3]. A total of 44% of those hospitalized for HF suffer from diabetes. Previous studies have explored the possible mechanisms of the occurrence and development of ventricular remodeling caused by diabetes mellitus, including systemic insulin resistance, mitochondrial function, and altered cardiac metabolism. However, the particular mechanisms’ elucidation is not yet complete, and a greater knowledge of the mechanistic processes might lead to the development of novel pharmacological targets for DCM therapy.

Oxidative stress has been considered a primary risk factor for diabetes-related complications. Although a minimal amount of reactive oxygen species (ROS) are required for basic physiological functions, excessive reactive oxygen species production may lead to cell dysfunction in the cardiovascular and other systems [4]. Nicotinamide adenine dinucleotide phosphate (NADPH) oxidase is an enzyme that catalyzes the transfer of electrons from NADPH to molecular O_2_ and is one of the major generators of reactive oxygen species in the circulatory system [5]. In diabetes, hyperglycemia triggers a specific metabolic pathway including diacylglycerol (DAG), protein kinase C (PKC), and NOX, resulting in reactive oxygen species. In more detail, high blood glucose levels stimulate the synthesis of DAG formation, a physiological activator of PKC. This phosphorylates the subunit of NADPH-oxidase, leading to its activation [6]. The increased NOX activation and excessive reactive oxygen species generation results in cardiac oxidative stress and inflammation, which leads to cardiac fibrosis, cardiac hypertrophy, and left ventricular diastolic dysfunction. Our previous studies have suggested that the mitochondrial capability to cope with reactive oxygen species plays a crucial role in diabetic-related cardiac remodeling [7,8,9]. However, their detailed pathological mechanisms are still unclear. Meanwhile, it was reported that the mitochondrial sirtuin SIRT3 may sequester forkhead box O3a (FOXO3a) in the nucleus, increasing the transcription of Mn superoxide dismutase (SOD2) and enhancing mitochondrial reactive oxygen species scavenging capabilities [10]. In the present study, we tested the hypothesis that hyperglycemia generates reactive oxygen species by activating NOX in ventricular myocytes, which then mediates adverse ventricular remodeling, and increases the vulnerability to DCM. Apocynin treatment corrects the aforementioned abnormality by suppressing reactive oxygen species overproduction and restoring SIRT3/FOXO3a expression.

## 2. Materials and Methods

### 2.1. Animals

Aged 6–8 weeks, male C57BL/6 mice were used. All mice were kept in a controlled environment (20 2 C, 12 h/12 h light/dark cycle) with unrestricted access to water. Type 2 diabetes mellitus was induced using a high-fat diet (HFD, 60 kcal% fat, 20 kcal% carbohydrate, and 20 kcal% protein) combined with low-dose streptozotocin (STZ). All mice were assigned randomly to the following 3 groups by a random number table: control, diabetes, and diabetes with apocynin (*n* = 6 each group).

The control group received a normal chow diet for the duration of the study, while the diabetes and diabetes with apocynin groups received a high-fat diet. After 4 weeks, the diabetes and diabetes with apocynin groups were produced by intraperitoneal injection of STZ (60 mg/kg; Sigma-Aldrich, St. Louis, MO, USA) in 0.1 M citrate buffer. The citrate buffer vehicle was injected into the mice in the control group. At 72 h after the STZ injection, the fasting blood glucose levels (FBG) of the HFD group were evaluated. The induction of diabetes was deemed effective if the mice had FBG > 11 mmol/L; those mice were employed in further experiments. Having successfully induced type 2 diabetes, diabetes with apocynin was administered by daily gavage of apocynin (40 mg/kg; Sigma, Saint Louis, MO, USA) dissolved in corn oil for 8 weeks, while the other groups simply received corn oil. All animals were sacrificed for subsequent experiments at 12 weeks. The investigation conformed to the Guide for the Care and Use of Laboratory Animals by the US National Institutes of Health. The study protocols and use of animals were approved by the Experimental Animal Administration Committee of Tianjin Medical University (TMUaMEC 2019004).

### 2.2. Echocardiography

All mice underwent transthoracic echocardiography utilizing a Vevo 2100 system with an MS400 linear array transducer (VisualSonics, Toronto, ON, Canada). Briefly, mice were sedated with 2% isoflurane and kept warm on a platform heated to 37 °C. The chest hairs were eliminated with depilatory lotion, and acoustic coupling gel was then applied. At the level of the mid-papillary muscle, an average of 10 cardiac cycles of conventional 2D and m-mode short axis were studied. Left ventricular ejection fraction and dimensions were calculated by using a modified Quinone method [9].

### 2.3. Electrophysiological Study

The mice were intraperitoneally injected with 1.5% tribromoethanol (50 mg/kg) for anesthesia. On the epicardial surface of the left ventricle, a 6 × 6 electrode microelectrode (electrode impedance: 1.5-1.7Q, PA03606060101, multielectrode probe array) was used to record epicardial activating electrical mapping. Data were recorded by multichannel systems (EMS64-USB-1003, MappingLab Ltd., Oxford, UK). Acquired activation waveforms were amplified by a filter amplifier and then communicated to a computer. All activation times was digitized and then applied to the creation of activation maps. The activation times were calculated as the point of maximal negative slope of activation waveforms. EMapScope 4.0 software (MappingLab Ltd., Oxford, UK) was used to figure out the conduction velocity (CV), the inhomogeneity index, and the absolute inhomogeneity.

### 2.4. Blood Pressure

Mice were put in animal holders on a far-infrared warming platform, and their body temperatures were kept at 37 °C. The blood pressure of conscious mice was measured utilizing volume pressure recording sensor equipment (BP-98-AL, Softron, Tokyo, Japan). After a time of stabilization, systolic blood pressure (SBP), diastolic blood pressure (DBP), and mean blood pressure (MBP) were meticulously monitored.

### 2.5. Cell Culture, siRNA Transfection, and Treatment

The H9c2 cell line (Cellcook Biotech Co., Ltd., Guangzhou, China) was used for in vitro studies and maintained in DMEM media with 1.0 g/L Glucose (10014CM, Corning, New York, NY, USA) containing 10% fetal bovine serum (FBS, Gibco, Waltham, MA, USA). In the following tests, H9c2 cells were cultured in 2% fetal bovine serum (FBS) media for 12 h before treatment. The cells were pretreated with 100 μM apocynin (Sigma, Saint Louis, MO, USA) for 1 h and stimulated with 33 mM high glucose (HG) for 36 h. The cells were divided into the following groups at random: NC, HG, and HG + APO groups.

When cells reached up to 70% confluence, H9c2 cells were seeded into six-well plates and transfected with control siRNA or SIRT3 siRNA (GenePharma, Suzhou, China) for 24 h using a Lipofectamine 3000 transfection reagent (Thermo Fisher Scientific, Waltham, MA, USA). Then, H9c2 cells were treated with 100 μM apocynin for the next 24 h. Cells were rinsed with PBS after being exposed to apocynin for further measurements.

### 2.6. Histology

The tissues were cut into 5 μm cross sections, then stained with hematoxylin and eosin (HE) to observe the morphological changes and Masson’s trichrome stain to evaluate interstitial fibrosis. Image-Pro Plus 6.0 software (Media Cybernetics Co., Ltd., Carpenteria, CA, USA) was used to examine cell morphology and observe the volume percentage of ventricular interstitial collagen.

Harvested hearts were fixed and embedded in optimal cutting temperature compound (OCT) (VWR, PA) for immunofluorescence staining. Then, the hearts were cut into 5 μm cross sections and stained with primary antibody for Ac-FOXO3a (Lys271) (1:200; AF3771, Affinity) overnight at 4 °C. Alex 488-conjugated goat anti-rabbit antibody (1:200, SA00013-2, Proteintech) was used as a secondary antibody. DAPI was used to stain the nuclei. Images were acquired under an Olympus inverted microscope (IX81, Tokyo, Japan). ImageJ was used to calculate the intensity of the fluorescent light.

### 2.7. ROS Detection

For ROS detection, dihydroethidium (DHE) dye is utilized. Frozen sections of ventricular tissue and H9c2 cells measuring 5 μm thick were stained with DHE (10 μmol/L, Molecular Probes, Sigma, Saint Louis, MO, USA) and incubated at 37 °C for 30 min in a light-protected humidifying box. An Olympus inverted microscope (IX81, Olympus Corporation, Tokyo, Japan) was used to capture images of ventricular tissue. Flow cytometry (FACSVerse, BD, Franklin Lake, NJ, USA) was used to evaluate H9c2 cells within 1 h. The stripe was quantified using Image J software (NIH Image, Bethesda, MD, USA) [11].

### 2.8. Western Blot Analysis

Proteins were extracted from snap-frozen ventricular tissue and the cultured H9c2 cell line by RIPA lysis buffer. Protein concentrations were measured by the bicinchoninic acid (BCA) protein assay reagent kit (Thermo Scientific, Waltham, MA, USA). Then, 30 μg of each protein was separated on SDS-PAGE and electrotransferred onto PVDF membranes, blocked with TBST containing 5% non-fat dry milk, and blots on membranes incubated with antigen and antibody complexes were detected by an ECL protocol with horseradish peroxidase-conjugated IgG as secondary antibodies.

The primary antibodies used were as follows: β-actin (1:3000, AB0035, Abways), mitochondrial transcription factor A (TFAM, 1:1000, ab131607, Abcam), mitofusin 2 (MFN2, 1:1000, D2D10, Cell Signaling Technology), α-smooth muscle actin (α-SMA, 1:1000, ab7817, Abcam), peroxisome proliferator-activated receptor gamma coactivator 1-alpha (PGC1-α, 1:1000, ab106814, Abcam), Manganese Superoxide Dismutase (Mn-SOD, 1:1000, D3X8F, Cell Signaling Technology), Manganese Superoxide Dismutase (acetyl K68) (Mn-SOD (acetyl K68), 1:1000, ab137037, Abcam), Sirtuin 3 (SIRT3, 1:1000, D22A3, Cell Signaling Technology), and Forkhead box class O 3a (FOXO3a, 1:1000, 75D8, Cell Signaling Technolog). β-actin was evaluated as a loading control. The reactions were visualized using Tanon 5200 Multi Chemiluminescent Imaging System (Tanon Science & Technology Co., Ltd., Shanghai, China). Image J software (NIH Image, Bethesda, MD, USA) was used further to quantify the stripe.

### 2.9. Statistical Analysis

Data were presented as mean ± standard deviation (SD). The normal distribution of the results was checked by the Kolmogorov-Smirnov (KS) test. Data conforming to a normal distribution were statistically analysed using the one-way analysis of variance (ANOVA) followed by the Least Significant Difference (LSD) test. Non-parametric tests were used for non-normally distributed data. All data were analyzed using GraphPad Prism 8.4.0. The criterion for statistical significance was *p* < 0.05.

## 3. Results

### 3.1. Apocynin Improves Cardiac Function in Diabetic Mice

**Figure 1:**
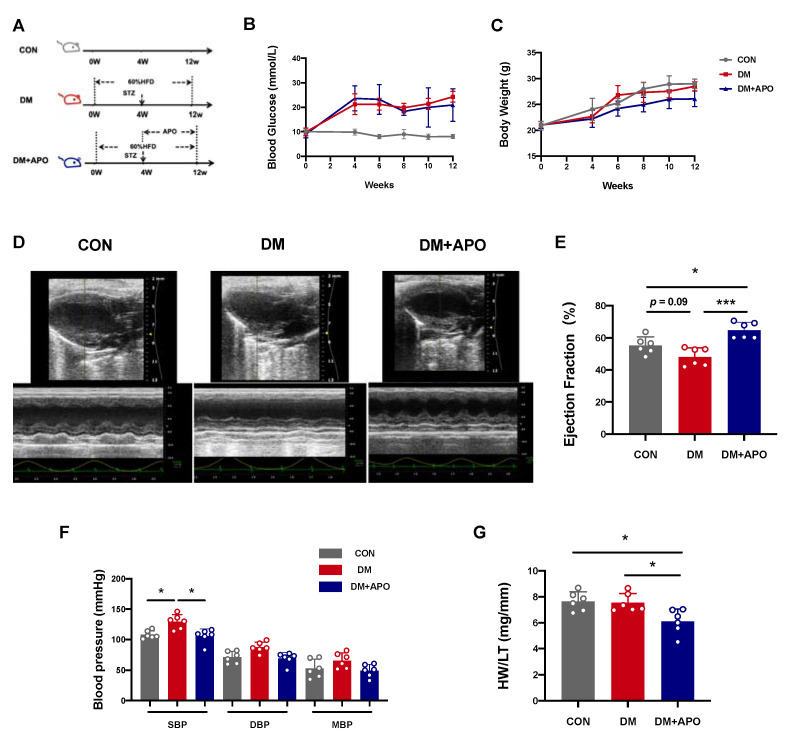
Apocynin (APO) improves cardiac function in diabetic mice. (**A**) C57BL/6 mice were induced with type 2 diabetes by high-fat diet combined with low-dose STZ. The DM+APO group was given APO (40 mg/kg) dissolved in corn oil by daily gavage. (**B**,**C**) Blood glucose and body weight in the 3 groups. (**D**) Representative two-dimensional and M-mode of echocardiographic images. (**E**) Analysis results of left ventricular ejection fraction (LVEF). (**F**) Results of hemodynamic studies (SBP, DBP, and MBP). (**G**) Ratio of heart weight (HW) to tibial length (TL) of mice in each group. Data are mean ± SD; *n* = 6 mice per group; * *p* < 0.05, *** *p* < 0.001.

We successfully established a model of type 2 diabetes using a high-fat diet (HFD) in combination with STZ. Mice in the diabetic and apocynin groups received APO dissolved in corn oil (40 mg/kg) by daily gavage, while the other group received only corn oil daily. We performed the following experiments on all mice after 8 weeks of treatment (Figure 1A). Compared to the control group, blood glucose levels were slightly elevated in the diabetes group (21.23 ± 4.28 mmol/L, *p* < 0.01) and the diabetes with apocynin group (23.63 ± 5.12 mmol/L, *p* < 0.01) after injecting STZ. Blood glucose levels did not vary between the two diabetes groups. Randomized blood glucose levels were maintained at >11 mmol/L throughout the 8 weeks of the study (Figure 1B). Body weight had no significant difference among the three experimental groups (Figure 1C).

Table 1 displays the echocardiographic parameters of the three research groups. Representative two-dimensional and M-mode echocardiography images are shown in Figure 1D. Echocardiography analysis revealed a moderate decrease in ejection fraction (EF) in the diabetes group compared to the control group (55.20 ± 5.33 vs. 48.13 ± 5.62, *p* = 0.09, Figure 1E). Moreover, SBP in the diabetes group increased compared to the control group (129.39 ± 11.76 vs. 107.78 ± 6.44, *p* < 0.05). The diabetic state alone also slightly increased the ratio of heart weight (HW) to tibial length (TL) (HW/TL) (Figure 1G). All of the above abnormalities were prevented by apocynin treatment (*p* < 0.05).

### 3.2. Apocynin Improves the Ventricular Structural and Electrical Modeling in Diabetic Mice

**Figure 2:**
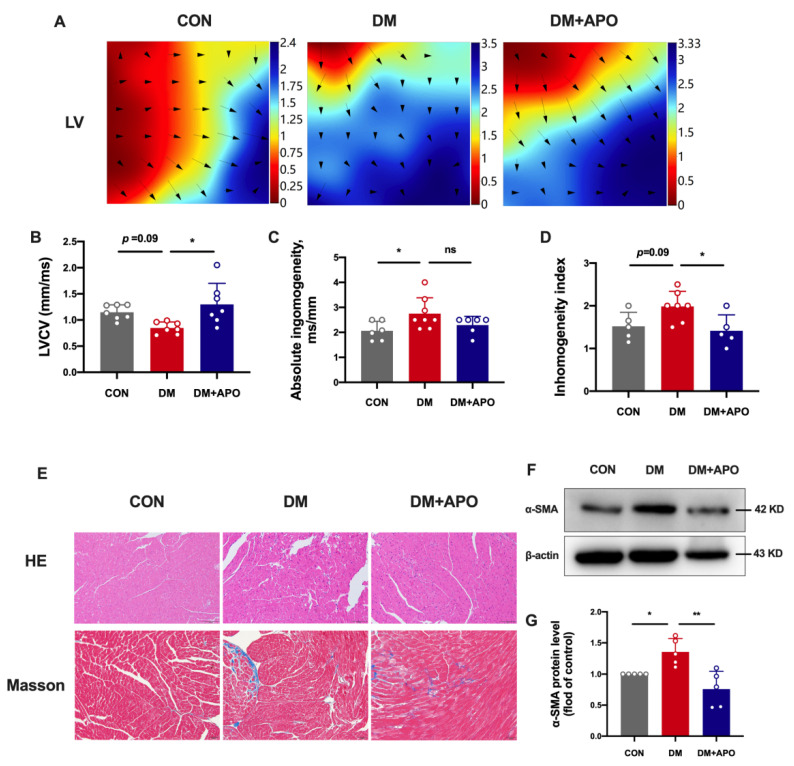
Apocynin improves the ventricular structural and electrical modeling in diabetic mice. (**A**) Recorded representative epicardial activation mapping of left ventricular (LV) in the 3 groups. (**B**–**D**) Mean left ventricular conduction velocity (LVCV), absolute inhomogeneity, and inhomogeneity index of LV between each group. (**E**) Representative images of HE and Masson’s trichrome staining. (**F**) Expression of α-SMA among the 3 groups. (**G**) Quantification of α-SMA in (F). Data are mean ± SD; *n* = 5–8 mice per group; * *p* < 0.05, ** *p* < 0.01.

Figure 2A–D shows the results of left ventricle epicardial mapping. The diabetes group had a lower left ventricular conduction velocity (*p* = 0.09), higher left atrial absolute inhomogeneity (*p* < 0.05), and higher conduction inhomogeneity index (*p* = 0.09). These changes were averted by apocynin therapy (*p* < 0.05, *p* = 0.2, and *p* < 0.05, respectively).

H&E and Masson’s staining demonstrates morphological changes in the left ventricle. Cardiomyocytes in the diabetes group exhibited a higher cross-sectional area and disorganized myofilaments. Cardiomyocyte hypertrophy and myocardial fibrosis improved after apocynin treatment (Figure 2E). Meanwhile, apocynin attenuated hyperglycemia-induced α-SMA expression (*p* < 0.01) (Figure 2F,G).

### 3.3. Apocynin Decreases ROS and Improves Mitochondrial Function of Diabetic Cardiomyopathy

**Figure 3:**
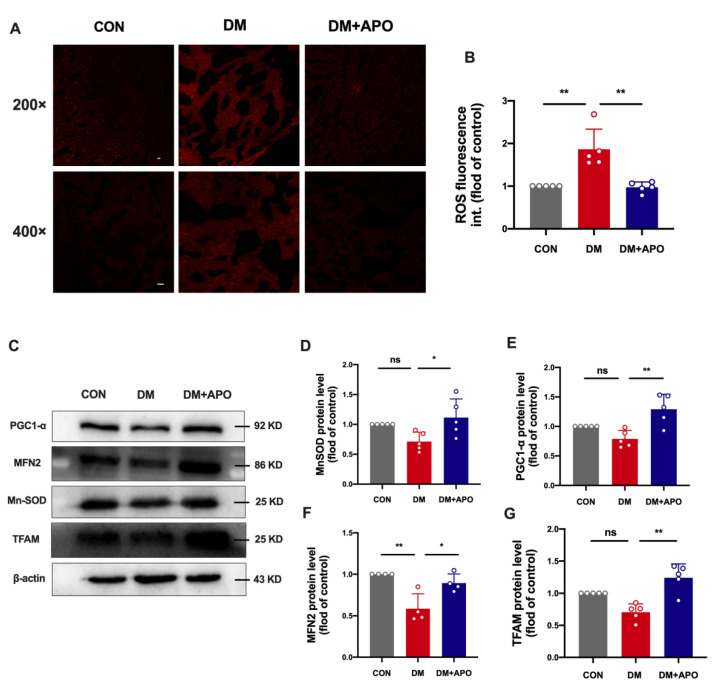
Apocynin decreases reactive oxygen species (ROS) and improves mitochondrial function of diabetic cardiomyopathy. (**A**,**B**) Production of ROS in ventricular tissues. Scale bar, 20 μm. (**C**) Expression of PGC1-α, Mn-SOD, TFAM, and MFN2 among the 3 groups. (**D**–**G**) Quantification of PGC1-α, Mn-SOD, TFAM, and MFN2 in (C). Data are mean ± SD; *n* = 5 mice per group; * *p* < 0.05, ** *p* < 0.01.

Fluorescence of DHE in ventricular tissue was evaluated in each experimental group (Figure 3A). The ROS concentration in situ was significantly higher in the diabetes group compared to the control group (*p* < 0.01), while the apocynin treatment reversed the oxidative stress in ventricular tissue (*p* < 0.01, Figure 3B).

To determine the mitochondrial function in the three groups, we evaluated the expression of mitochondria-associated proteins of ventricular tissue and found that the expression levels of PGC1-α, Mn-SOD, TFAM, and MFN2 showed a decreasing trend in the diabetes group compared to the control group (Figure 3C–G). Encouragingly, expression levels of these mitochondria-associated proteins significantly increased after apocynin treatment (Figure 3C–G).

### 3.4. Apocynin Regulates SIRT3/FOXO3a Signaling Pathway in Diabetic Cardiomyopathy

**Figure 4:**
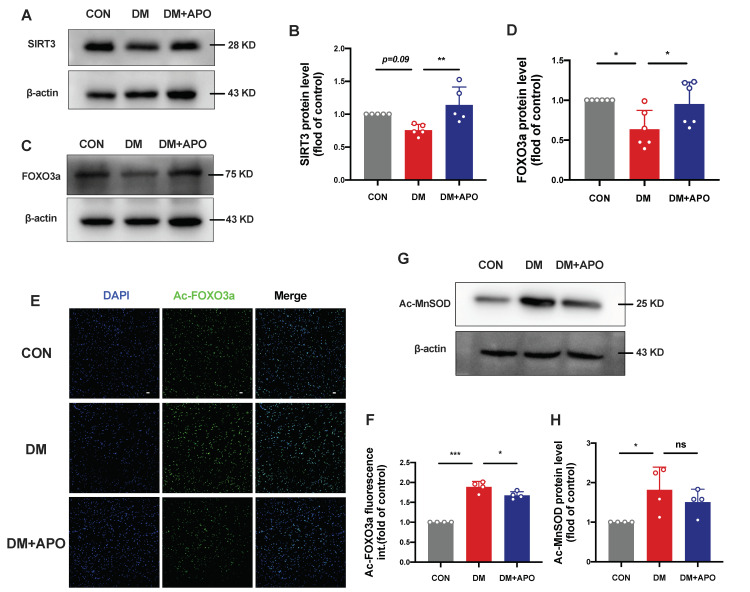
Apocynin regulates SIRT3/FOXO3a signaling pathway in diabetic cardiomyopathy. (**A**) Protein level of SIRT3 detected by Western blot analysis. (**B**) Quantification of SIRT3 in (A). (**C**) Expression of FOXO3a in ventricular tissues. (**D**) Quantification of FOXO3a in (C). (**E**) Representative confocal microscopy images of immunofluorescence stained for Ac-FOXO3a and DAPI. (**F**) Ac-FOXO3a fluorescence intensity quantification. Scale bar, 20 μm. (**G**) Expression of Ac-MnSOD in ventricular tissues among 3 groups. (**H**) Quantification of Ac-MnSOD in (G). Data are mean ± SD; *n* = 5 mice per group; * *p* < 0.05, ** *p* < 0.01, *** *p* < 0.001.

There is emerging evidence that SIRT3 resides primarily in the mitochondria and has been shown to bind and deacetylate several metabolic and respiratory enzymes that regulate important mitochondrial functions [12], and recent studies show that SIRT3 plays vital role in cardiovascular physiology and pathology [13]. Here, we hypothesized that apocynin regulates mitochondrial function by improving SIRT3-mediated deacetylation of mitochondrial proteins. Compared to the control group, the expressions of SIRT3 showed a decreased trend in the diabetes group (*p* = 0.09, Figure 4A,B), and apocynin treatment significantly reversed SIRT3 expressions (*p* < 0.01). Meanwhile, Mn-SOD acetylation at K68 significantly increased in the diabetes group compared to the CON and APO groups (*p* < 0.05, *p* = 0.5, respectively, Figure 4G,H). Since FOXO3 is a direct target of SIRT3 and functions as a forkhead transcription factor to govern cellular responses to diverse stress, we explored the total and acetylated FOXO3a expression. Hyperglycemia suppressed FOXO3a expression and induced FOXO3a acetylation at Lys271 on immunofluorescent staining (*p* < 0.01, *p* < 0.0001, respectively, Figure 4C–F).

### 3.5. Apocynin Attenuates Oxidative Stress via SIRT3/FOXO3a Pathway in H9c2 Cells

**Figure 5:**
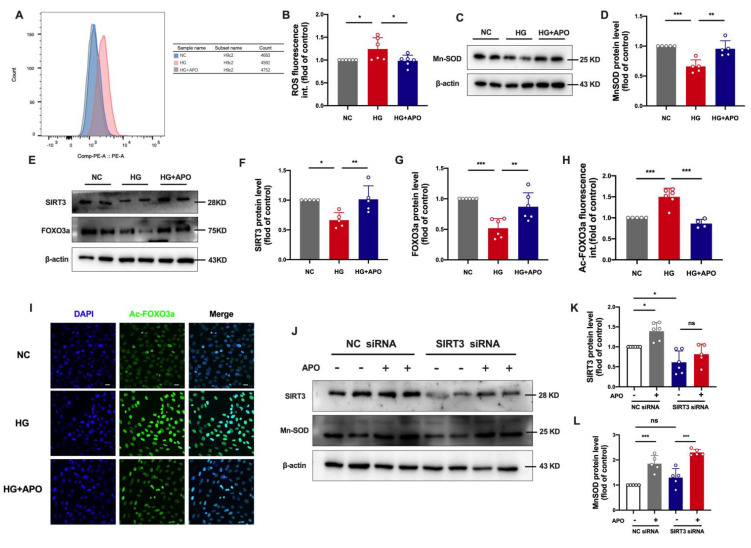
Apocynin (APO) attenuates oxidative stress via the SIRT3/FOXO3a pathway in H9c2 cells. (**A**) H9c2 cells were exposed to normal glucose (NC) or high glucose (HG) or HG with APO for 36 h. Flow cytometry detected H9c2 cells stained with Dihydroethidium (DHE). (**B**) Quantification of ROS by DHE intensity in (A). (**C**) Mn-SOD protein level in H9c2 cells detected by Western blot analysis. (**D**) Quantification of Mn-SOD protein level in (C). (**E**) The protein levels of SIRT3 and FOXO3a in each group. (**F**,**G**) Quantification of SIRT3 and FOXO3a protein level in (E). (**H**) Ac-FOXO3a fluorescence intensity quantification in (I). Scale bar, 20 μm. (**I**) Representative confocal microscopy images of immunofluorescence staining for Ac-FOXO3a and DAPI in H9c2 cells exposed HG with or without APO. (**J**) Expression of SIRT3 and Mn-SOD in NC siRNA and SIRT3 siRNA with or without APO. (**K**,**L**) Quantification of SIRT3 and Mn-SOD protein level in (J). Data are mean ± SD; *n* = 4 for 6 independent experiments; * *p* < 0.05, ** *p* < 0.01, *** *p* < 0.001.

We next explored the role of apocynin in oxidative stress caused by SIRT3 diminution. Downregulation of SIRT3 expression by specific siRNA led to a reduction of Mn-SOD in H9c2 cells. Apocynin would increase SIRT3 expression in both NC and SIRT3 siRNA group. Meanwhile, apocynin promoted significant recovery of Mn-SOD expression in the SIRT3 siRNA group (Figure 5J–L). These results indicate that apocynin might attenuate HG-induced mitochondrial dysfunction by improving the SIRT3/FOXO3a pathway.

## 4. Discussion

**Figure 6:**
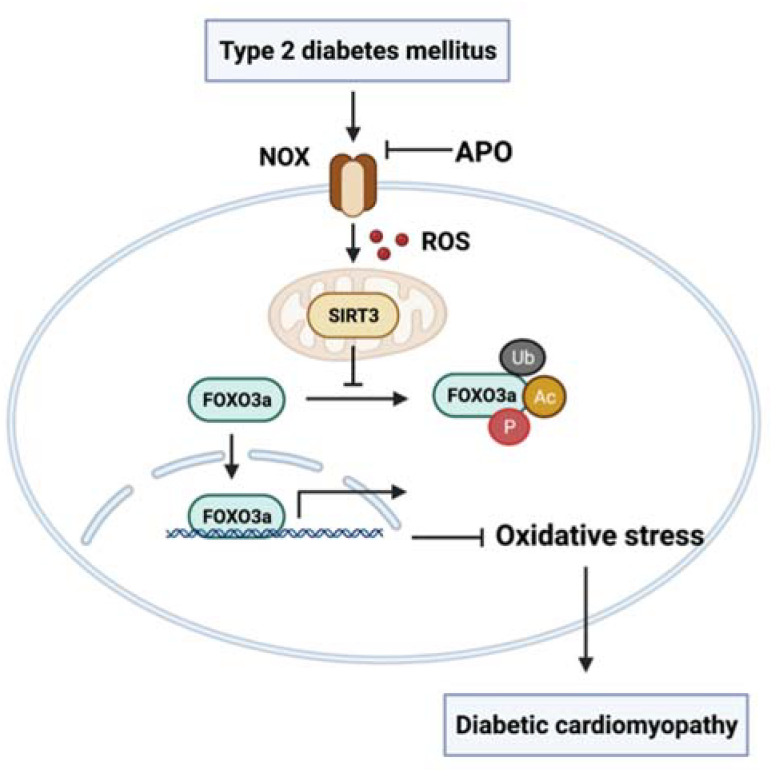
Within diabetic ventricles, NADPH oxidase (NOX) is an enzyme that plays a significant part in the development of oxidative stress. Apocynin (APO) can improve myocardial reactive oxygen species (ROS) overproduction and mitochondrial function through recovery of the SIRT3/FOXO3a pathway, thereby reducing the incidence of diabetic cardiomyopathy. It is possible that this substance will be developed into a medication for use in the clinical management of diabetic ventricular remodeling.

The following are the key conclusions: (1) We observed ventricular mitochondria-associated protein (PGC1-α, Mn-SOD, TFAM, and MFN2) dysregulation and excessive reactive oxygen species production in the diabetes group. (2) These changes were associated with a decreased LVEF and increased left ventricular interstitial fibrosis, a higher conduction inhomogeneity index, and a greater rate of inducible DCM. (3) By suppressing reactive oxygen species overproduction and restoring SIRT3/FOXO3a expression, apocynin therapy may be able to totally or substantially correct the aforementioned abnormalities (Figure 6).

Diabetes mellitus is one of the most common chronic diseases in the world [14,15]. Diabetic cardiomyopathy (DCM) is a specific cardiac manifestation of diabetic patients characterized by left ventricular hypertrophy and diastolic dysfunction in the early phase up to overt heart failure with diminished systolic function in the advanced stage [16]. In diabetes mellitus, hyperglycemia, systemic insulin resistance, and altered cardiac metabolism are all major clinical abnormalities that play a role in the DCM etiology. The Framingham Heart Study showed that diabetic people have twice the risk of HF in men and five times in women as nondiabetic patients [3], and 44% of those hospitalized for HF suffer from diabetes [3]. Diabetes mellitus has been considered one of the main mitochondrial diseases and oxidative stress is a primary risk factor for diabetes-related complications [17,18,19,20]. Its effects on diabetic neuropathies and nephropathies are well-known, and recent evidence suggests that oxidative stress may also contribute to diabetes-related heart disease, including atrial fibrillation (AF), ventricular arrhythmias, and DCM [9,11,21,22,23]. The underlying mechanisms of the action of oxidative stress in DCM are not well-understood but include effects on cellular redox-sensitive signaling pathways, mitochondrial dysfunction, and generation of reactive oxygen species. The consequence of hyperglycemia-mediated reactive oxygen species overproduction is the triggering of the biological abnormalities in diabetic complications, which is consistent with our previous findings [9,11,24]. In our present study, we found that hyperglycemia caused excessive reactive oxygen species generation through disrupting mitochondrial biogenesis (via PGC1α and TFAM), mitochondrial fusion (via MFN2), and the reactive oxygen species scavenging process (via Mn-SOD) in the ventricular tissue of mice with type 2 diabetes.

Nicotinamide adenine dinucleotide phosphate (NADPH) oxidase, also known as non-phagocytic cell oxidase (NOX), is a major generator of reactive oxygen species in cells. They act as electron transport chains across membranes, reducing molecular oxygen to O_2_-utilizing NADPH as an electron source [25]. Our previous research has shown that NOX activation by diabetes mellitus impairs the ultrastructure of atrial mitochondria and causes respiratory dysfunction in rabbits. However, the inhibitory effect of apophynin on NOX and Rac1 expression can improve diabetes-related atrial remodeling [11]. NOX is activated when it is translocated to the membrane and then combines with p22phox, p40phox, p47phox, and p67phox, which are required for NOX to serve its purpose. Prior research has demonstrated that NOX activity and expression of certain subunits are elevated in diabetes mellitus. Through activation of protein kinase C and p47phox, glucose enhanced NOX2 activity in monocytes, resulting in reactive oxygen species production [26]. Apocynin is a methoxy-substituted catechol produced by plants that has been extensively utilized as a non-specific NOX inhibitor [5,27]. The active form of diapocynin has the ability to oxidize thiol groups, which prevents the translocation of cytosolic p47phox and p67phox to the membrane and disrupts the formation of enzyme complexes [28,29]. Our study employs apocynin as a powerful NOX inhibitor to achieve a reduction in reactive oxygen species generation and minimize reactive oxygen species production. The study found that LVEF depression, myocardial fibrosis, and high conduction heterogeneity caused by diabetes were all helped by apocynin treatment, which stopped ROS from making too much.

Mitochondria are assumed to be the primary source of oxidative stress during oxidative phosphorylation. Oxidative stress in mitochondria may contribute to additional mitochondrial dysfunction by triggering ROS-dependent ROS release in multiple tissues [30]. This may lead to Δψ collapse, mitochondrial enlargement, cytochrome c release and apoptosis. Next, we investigated whether inhibiting ROS generation using apocynin may enhance mitochondrial homeostasis, which is critical for its quantity and function. Firstly, mitochondrial biogenesis is a complicated process. Peroxisome proliferator-activated receptor gamma coactivator 1-alpha (PGC-1α) is the primary activator that regulates the mitochondrial biogenesis signaling pathway through its downstream mediator transcription Factor A (TFAM). The PGC-1/TFAM signaling pathway is involved in mitochondrial biogenesis in various disorders. According to Li et al., activating the PGC-1/TFAM signaling pathway could result in mitochondrial DNA and protein synthesis [31]. Zhang et al., also demonstrated that alogliptin could upregulate the PGC-1α/TFAM signaling pathway and prevent diastolic dysfunction in diabetic rabbits via reversing mitochondrial membrane depolarization and mitochondrial swelling [7]. Secondly, mitochondrial fusion and fission are essential for cellular function [32]. Accumulated findings imply that low mitofusin2 (MFN2) levels in diabetic hearts disturb the balance of fission and fusion dynamics, hastening the onset of DCM [33]. According to our recent study, under situations of endoplasmic reticulum (ER) stress, silencing MFN2 lowers Ca^2+^ transport from the endoplasmic reticulum to the mitochondria, ameliorating mitochondrial reactive oxygen species generation and oxygen consumption and protecting cells from mitochondrial malfunction and ER-mediated cell death [24]. Finally, antioxidant enzymes scavenge excess reactive oxygen species and reduce cellular oxidative stress to maintain redox equilibrium. Superoxide dismutase (SOD) is the first and most important antioxidant enzyme family that scavenges reactive oxygen species, particularly O_2_^−^, and its primary function is to catalyze the disproportionation of O_2_^−^ to H_2_O_2_ and O_2_ [34]. As demonstrated by our present study, diabetes causes a dysregulation in the expression of mitochondria-associated proteins in the ventricular myocardium, including PGC-1 α, Mn-SOD, TFAM, and MFN2. This could be because diabetes promotes hyperglycemia, which activates NOX in the cardiomyocyte. This then leads to an increase in NOX activity and the production of reactive oxygen species, which further contributes to mitochondrial dysfunction. Apocynin can ameliorate this functional impairment by inhibiting NOX.

Another important finding of the present study is that the downregulated SIRT3/FOXO3a pathway caused by NOX activation in turn results in an increased generation of reactive oxygen species, aggravating the diabetes-related ventricular remodeling. The mitochondrial SIRT3 is a NAD^+^-dependent protein deacetylase and is a key class III histone deacetylase that eliminates mitoROS, inhibits apoptosis, and prevents the formation of cancer cells [35]. SIRT3 has a stronger deacetylase activity than SIRT4 or SIRT5; hence, it plays a significant role in mitochondrial biology and pathophysiology to cope with reactive oxygen species [36]. SIRT3 can directly regulate the activity of the key superoxide scavenger (MnSOD) by deacetylation [13]. SIRT3-deficient animals had diminished MnSOD activity in the heart [37]. In in vitro studies of SIRT3^-/+^ mouse embryonic fibroblasts, SIRT3 expression deacetylated MnSOD, increased its activity, and decreased reactive oxygen species in the mitochondria and cells [38]. Moreover, FOXO3, a mitochondrion-located transcription factor, is also shown as a direct target of SIRT3. SIRT3-induced deacetylation of FOXO3 lysine at K271 and K290 plays an indispensable role in mitochondrial function by coordinating mitochondrial fusion/fission processes, mitochondrial biogenesis, and mitophagy [39]. In the present paper, under HG conditions, SIRT3 levels were reduced, which led to FOXO3a acetylation at Lys271 as well as cellular reactive oxygen species excess. Apocynin promoted SIRT3 and MnSOD expression in both NC and SIRT3 siRNA groups.

It’s important to be aware of our study’s limitations. Firstly, due to the limitations of equipment, the activation of NADPH oxidase was not investigated. Secondly, only the FOXO3a acetylation at Lys271 was tested by immunofluorescence staining; we did not explore FOXO3a acetylation at K290 and its specific mechanisms. Finally, future research should examine the preventive effects of various apocynin dosages, which were not examined in this study.

## 5. Conclusions

In summary, within diabetic ventricles, NADPH oxidase is an enzyme that plays a significant part in the development of oxidative stress. Apocynin can improve myocardial reactive oxygen species overproduction and mitochondrial function through the recovery of the SIRT3/FOXO3a pathway, thereby reducing the incidence of diabetic cardiomyopathy. It is possible that this substance will be developed into a medication for use in the clinical management of diabetic ventricular remodeling.

## Figures and Tables

**Table 1:** Echocardiographic parameters of mice in the three groups.

	CON Group (*n* = 6)	DM Group (*n* = 6)	DM + APO Group (*n* = 6)
HR (bpm)	522.33 ± 46.04	528.33 ± 57.49	552.00 ± 50.53
IVS (mm)	0.83 ± 0.17	0.95 ± 0.12	0.86 ± 0.15
LVEDD (mm)	4.02 ± 0.24	3.71 ± 0.28	3.61 ± 0.18
LVESD (mm)	2.92 ± 0.18	2.47 ± 0.22 *	2.61 ± 0.19
LVPW (mm)	0.88 ± 0.26	0.97 ± 0.17	0.89 ± 0.18
E/A	2.36 ± 0.76	1.53 ± 0.49	1.77 ± 0.56
LVEF (%)	55.20 ± 5.33	48.13 ± 5.6	64.68 ± 5.04 *

HR: Heart rate; IVS: interventricular septal thickness; LVEDD: left ventricular end-diastolic dimension; LVESD: left ventricular end-systolic dimensions; LVPW: left ventricular posterior wall thickness; LVEF: left ventricular ejection fraction. Data are mean ± SD; *n* = 6 mice per group; * *p* < 0.05.

## Data Availability

All of the data are contained within the article.
